# Correlation of TyG-BMI and TyG-WC with severity and short-term outcome in new-onset acute ischemic stroke

**DOI:** 10.3389/fendo.2024.1327903

**Published:** 2024-05-23

**Authors:** Xin-Rui Yu, Jing-Lu Du, Mei Jiang, Yuan Ren, Fu-Liang Zhang, Fan-Li Kong, Feng-E. Li

**Affiliations:** ^1^ Postgraduate Department, School of Clinical Medicine, Beihua University, Jilin, China; ^2^ Department of Neurology, Zhujiang Hospital, Southern Medical University, Guangzhou, China; ^3^ Department of Neurology, The First Hospital of Jilin University, Changchun, China; ^4^ Department of Pathophysiology, School of Basic Medicine, Beihua University, Jilin, China; ^5^ Neurology Department, the Affiliated Hospital of Beihua University, Jilin, China

**Keywords:** triglyceride-glucose index, body mass index, waist circumference, ischemic stroke, neurological deficits, outcome, insulin resistance

## Abstract

**Objectives:**

To research the connection between the indexes of the indexes of triglyceride-glucose (TyG) combined with obesity indices and the initial neurological severity and short-term outcome of new-onset acute ischemic stroke.

**Methods:**

Data of patients with acute ischemic stroke admitted to the Stroke Ward of the Affiliated Hospital of Beihua University from November 2021 to October 2023, were collected. The two indexes were calculated by combining TyG and obesity indices: TyG-body mass index (TyG-BMI) and TyG-waist circumference (TyG-WC). The National Institute of Health Stroke Scale (NIHSS) was used to assess and group patients with neurological deficits within 24 hours of admission: mild stroke (NIHSS ≤5) and moderate-severe stroke (NIHSS >5). Short-term prognosis was evaluated using the modified Rankin Scale (mRS) at discharge or 14 days after onset of the disease and grouped: good outcome (mRS ≤2) and poor outcome (mRS >2). According to the quartiles of TyG-BMI and TyG-WC, the patients were placed into four groups: Q1, Q2, Q3 and Q4. Multi-factor logistic regression analysis was utilized to evaluate the correlation of TyG-BMI and TyG-WC with the severity and short-term outcome.

**Results:**

The study included 456 patients. After adjusting for multiple variables, the results showed that compared with the quartile 1, patients in quartile 4 of TyG-BMI had a reduced risk of moderate-severe stroke [Q4: OR: 0.407, 95%CI (0.185-0.894), P = 0.025]; Patients in quartiles 2, 3 and 4 of TyG-BMI had sequentially lower risk of short-term adverse outcomes [Q2: OR: 0.394, 95%CI (0.215-0.722), P = 0.003; Q3: OR: 0.324, 95%CI (0.163-0.642), P = 0.001; Q4: OR: 0.158, 95%CI (0.027-0.349), P <0.001]; Patients in quartiles 3 and 4 of TyG-WC had sequentially lower risk of moderate-severe stroke [Q3: OR: 0.355, 95%CI (0.173-0.728), P = 0.005; Q4: OR: 0.140, 95%CI (0.056-0.351), P <0.001]; Patients in quartiles 3 and 4 of TyG-WC had sequentially lower risk of short-term adverse outcomes [Q3: OR: 0.350, 95%CI (0.175-0.700), P = 0.003; Q4: OR: 0.178, 95%CI (0.071-0.451), P <0.001].

**Conclusions:**

TyG-WC and TyG-BMI were correlated with the severity and short-term outcome of new-onset acute ischemic stroke. As TyG-WC and TyG-BMI increased, stroke severity decreased and short-term outcome was better.

## 1 Introduction

Stroke, being one of the top causes of death and disability, has placed a rising load on the global medical system (1). The rates of disability and mortality in elderly stroke patients are expected to reach above 30% in an aging society, putting tremendous financial pressure and mental burden on the population (2). Although intravenous thrombolysis and intravascular therapy for acute ischemic stroke have grown quickly in recent years (3), it is still a great challenge to improve the efficacy of traditional drugs for large numbers of stroke patients who miss the recanalization of cerebral vessels. Therefore, it is particularly important to explore appropriate indicators for assessing the risk, severity, and prognosis of stroke early to better prevent and control the development of stroke.

Although obesity is recognized as a separate risk component for ischemic stroke (4), several studies have proven that obesity indicators in ischemic stroke individuals are frequently negatively associated with clinical prognosis, which is called the obesity paradox (5, 6). Higher BMI and WC were linked to less severe new-onset strokes and good outcomes in the ischemic stroke population, according to the Northern Manhattan Stroke Research (7, 8). A prospective cohort research revealed that triglycerides (TG) were positively linked with the prevalence of ischemic stroke in addition to obesity markers (9). Observational research conducted retrospectively, however, revealed that individuals with greater TG had fewer severe neurological symptoms and improved functional results (10, 11). It has been proposed that insulin resistance (IR) may be one of the mechanisms contributing to the obesity paradox in the outcome of ischemic stroke patients (12).

A prevalent pathological condition known as insulin resistance (IR) impairs the ability of cells to respond to insulin. There appears to be a significant positive relationship between IR and ischemic stroke, according to mounting research (13). The occurrence of IR is also strongly associated with obesity (14). The euglycemic-hyperinsulinemic clamp technique is the best practice for the diagnosis of IR currently, but its use is constrained by difficult procedures, substantial expenses, and moral dilemmas (15). TyG has been proposed as a straightforward substitute for IR (16) in recent years, with the advantages of low cost and easy accessibility. Song K et al. discovered that the combination of TyG and the obesity index can properly reflect the body’s IR level (17). It has been shown (18, 19) that for middle-aged and elderly adults experiencing a new-onset ischemic stroke, TyG-WC and TyG-BMI are significant predictors and have a higher predictive value than TyG. Nevertheless, the relationship remains unclear between the indexes of TyG combined with obesity indices and the severity and short-term prognosis of stroke. In this research, TyG-BMI and TyG-WC were calculated to explore the correlation between the indexes of TyG combined with obesity indices and the degree of neurological impairment and short-term outcome in new-onset acute ischemic stroke.

## 2 Materials and methods

### 2.1 Study population

**Figure 1:**
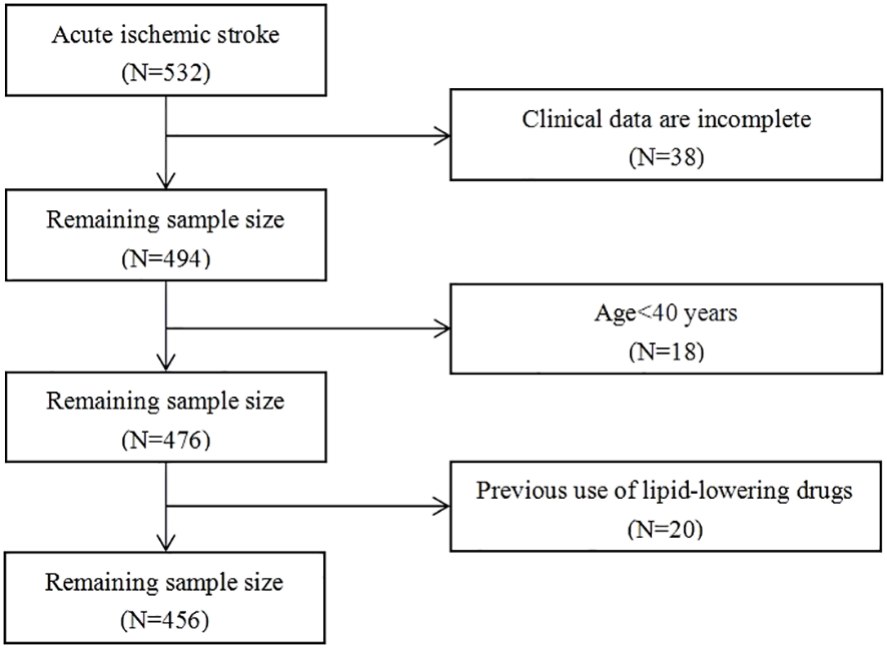
Flowchart for selecting this study participants.

Patients with acute ischemic stroke admitted to the Affiliated Hospital of Beihua University from November 2021 to October 2023, were retrospectively selected. This study was approved by the Medical Ethics Committee of the Affiliated Hospital of Beihua University. A total of 532 patients were included in this study, and 456 of them matched the screening criteria, as seen in the flow chart (**Figure 1**). The inclusion criteria: 1) the age at onset ≥40 years, 2) all patients and their families signed consent forms after being informed of the situation, 3) the study sample consisted of consecutive first-time acute ischemic stroke patients with onset ≤72 hours, and 4) brain CT or MRI scans were verified on all individuals. The exclusion criteria included the following: 1) previous cerebral infarction and cerebral hemorrhage, 2) suffering from autoimmune disease, severe infection or liver and kidney failure, 3) intravenous thrombolysis was given after admission, 4) lipid-lowering drugs were taken 1 month before admission, and 5) absence of clinical data.

### 2.2 Data collection and calculation

#### 2.2.1 Clinical features

Demographic characteristics of the enrolled patients were collected including gender, age; stroke risk factors included smoking, drinking, hypertension (HT), diabetes mellites (DM), coronary heart disease (CHD), atrial fibrillation (AF), and dyslipidemia (DL); clinical parameter included systolic pressure (SBP), diastolic blood pressure (DBP), height, weight, waist circumference (WC); laboratory tests included fasting blood glucose (FBG), triglyceride (TG), total cholesterol (TC), low-density lipoprotein cholesterol (LDL-C), high-density lipoprotein cholesterol (HDL-C), and post-admission treatment included antihypertensives, anticoagulants, antiplatelet agents, and statins.

#### 2.2.2 Related index calculation formula

1. TyG = Ln[(TG × FBG)/2] (20). Both TG and FBG are expressed in millimoles per liter.
2. BMI = Weight/Height^2^. The metric system uses meters for length and kilograms for weight.
3. TyG-BMI = TyG × BMI (21, 22).
4. TyG-WC = TyG × WC (23). WC is gauged in centimeters.
5. Unit conversion: TG: 1 mg/dl = 0.011 mmol/L, FBG = 1 mg/dl = 0.056 mmol/L.

#### 2.2.3 Measuring methods

All patients’ height and weight were recorded without shoes and in their underwear, the accuracy was 0.1 cm and 0.1 kg, respectively, and the instrument was a weight scale with a vertical fixed ruler (RGZ 120-RT, Wuxi Weighing Instrument Factory, China). Waist circumference was recorded with a soft ruler at the end of expiration at the middle point between the lower edge of the costal arch and the anterior superior iliac spine and was accurate to 0.1 cm (24). A manual sphygmomanometer (Desktop, Yuyue, China) was applied to measure blood pressure, and the patients were all rested for 15 min before measuring the blood pressure of both arms, and the blood pressure was repeated once after a 2-minute interval, and the mean value was recorded. Hematological indices were collected within 24 hours of admission and after 8 hours of fasting and sent to the laboratory for testing, and the instrument used was a fully automatic biochemical analyzer (AU5821, Beckman Coulter, China). The extended uncertainty (k=2) (%) of the indexes involved in this study is listed below, expressed in U%: FBG: 2.78%-3.24%, TG: 3.05%-3.31%, TC: 4.44%-5.04%, LDL-C: 9.04%-9.30%, HDL-C: 11.04%-11.30%. All the above values were tested by trained professionals and retested to ensure the accuracy of the tests.

### 2.3 Grouping

The National Institute of Health Stroke Scale (NIHSS) was used to score and group patients with neurological deficits within 24 hours of admission: mild stroke (NIHSS ≤5) and moderate-severe stroke (NIHSS >5). Short-term prognosis was evaluated using the modified Rankin Scale (mRS) at discharge or 14 days after onset of the disease and grouped into two groups: good outcome group (mRS ≤2) and poor outcome group (mRS >2). According to the quartiles of TyG-BMI and TyG-WC, the patients were placed into four groups: Q1, Q2, Q3 and Q4.

### 2.4 Diagnostic criteria for stroke risk factors

According to the World Health Organization, smokers were defined as individuals who had smoked continuously or cumulatively for more than 6 months. Alcohol drinkers were defined as cumulative alcohol intake of 98 grams or more per week in the past 6 months (25). Hypertension was considered systolic blood pressure ≥140 mmHg, diastolic blood pressure ≥90 mmHg, or being given antihypertensive medication (26). Fasting glucose of more than 7.0 mmol/L or current use of a glucose-lowering medication was considered to be diabetes mellitus (27). Self-reported medical history and electrocardiogram data were used to make the diagnosis of atrial fibrillation and coronary heart disease on admission.

### 2.5 Statistical method

SPSS 25.0 statistical software was utilized to examine the data. All measurement data were checked for normality. The data from the skewed distribution were reported as median [interquartile range (IQR)], and the Mann-Whitney U test was utilized to compare between two groups, and the Kruskal-Wallis H test was used for comparison between multiple groups of data. The frequency and percentage (%) of enumeration data were provided, and the χ^2^ test was performed to contrast groups. Multi-factor logistic regression analysis was performed using three models (model 1 unadjusted for confounders; models 2 and 3 adjusted for confounders), and the results were expressed as the odds ratio (OR) and 95% confidence intervals (CI). P <0.05 indicates statistical significance.

## 3 Results

A total of 532 patients with acute ischemic stroke were admitted during the study period, and after screening and exclusion, a total of 456 cases were included in the analysis, as shown in the flow chart (**Figure 1**). The median age of patients was 65 (IQR: 58-72 years) years, including 295 (64.7%) males and 161 (35.3%) females. Among the 456 patients, 350 (76.8%) had mild stroke, 106 (23.2%) had moderate to severe stroke, 297 (65.1%) had good outcomes, and 159 (34.9%) had poor outcomes.

### 3.1 Participants’ characteristics grouped by NIHSS score

As seen in **Table 1**, when comparing the two groups, there were statistically significant differences in age, smoker, AF, DL, SBP, DBP, TG, TyG-BMI as well as TyG-WC (P <0.05). Compared with the mild stroke group, moderate-severe patients had a higher AF proportion, age, SBP, DBP, and mRS score, and a less proportion of smokers, DL, and lower TG, TyG-BMI, and TyG-WC (P <0.05).

**Table 1:** Participants’ characteristics grouped by NIHSS score.

Variables	Total (N = 456)	Mild group (N = 350)	Moderate-severe group (N = 106)	Z/χ2	p
Demography					
Age (years)	65 (58–72)	65 (57–70)	67 (59–73)	-2.144	0.032
Gender (male)	295 (64.7)	229 (65.4)	66 (62.3)	0.357	0.550
Vascular risk factors					
Smoker	207 (45.4)	149 (42.6)	58 (54.7)	4.842	0.028
Alcohol drinker	161 (35.3)	122 (34.9)	39 (36.8)	0.133	0.715
HT	340 (74.6)	255 (72.9)	85 (80.2)	2.306	0.129
DM	172 (37.7)	136 (38.9)	36 (34.0)	0.830	0.362
CHD	77 (16.9)	56 (16.0)	21 (19.8)	0.842	0.359
AF	78 (17.1)	36 (10.3)	42 (39.6)	49.383	<0.001
DL	236 (51.8)	196 (56.0)	40 (37.7)	10.869	0.001
Blood pressure					
SBP (mmHg)	150 (135–165)	145 (135–160)	160 (140-175)	-4.271	<0.001
DBP (mmHg)	90 (80-100)	90 (80-100)	93 (80-105)	-3.121	0.002
Laboratory examination					
FBG (mmol/L)	5.95 (5.16-8.46)	5.99 (5.15-8.39)	5.90 (5.20-8.67)	-0.185	0.853
TG (mmol/L)	1.51 (1.09-2.07)	1.57 (1.12-2.19)	1.34 (0.95-1.73)	-3.369	0.001
TC (mmol/L)	4.71 (4.08-5.48)	4.77 (4.07-5.53)	4.63 (4.09-5.18)	-1.443	0.149
HDL-C (mmol/L)	1.15 (0.97-1.30)	1.14 (0.97-1.30)	1.16 (0.99-1.34)	-0.769	0.442
LDL-C (mmol/L)	2.71 (2.22-3.29)	2.72 (2.22-3.40)	2.63 (2.21-3.11)	-1.690	0.091
Involvement index					
TyG-BMI	225.19 (200.91-254.16)	231.22 (204.86-255.60)	211.55 (191.05-243.28)	-3.223	0.001
TyG-WC	829.00 (742.82-901.48)	839.34 (765.49-914.02)	761.72 (689.83-837.48)	-5.762	<0.001
**mRS**	2 (1-3)	2 (1-3)	4 (2-4)	-8.638	<0.001

Values are expressed as median (Q1–Q3) or n (%).

### 3.2 Participants’ characteristics grouped by mRS score in the mild stroke group

As seen in **Table 2**, among 350 patients with mild stroke, 255 (72.9%) had good outcomes and 95 (27.1%) had poor outcomes. When comparing the two groups, there were statistically significant differences in gender, age, smoker, alcohol drinker, DM, DL, FBG, TG, HDL-C, TyG-BMI as well as TyG-WC (P <0.05). Compared with the good outcomes group, patients with poor outcomes had higher age and HDL-C, and a less proportion of males, smokers, alcohol drinkers, DM, DL, and lower FBG, TG, TyG-BMI, and TyG-WC (P <0.05).

**Table 2:** Participants’ characteristics grouped by mRS score in the mild stroke group.

Variables	Total (N = 350)	Good outcome group (N = 255)	Poor outcome group (N = 95)	Z/χ2	p
Demography					
Age (years)	65 (57-70)	63 (56-69)	66 (59-73)	-2.930	0.003
Gender (male)	229 (65.4)	182 (71.4)	47 (49.5)	14.674	<0.001
Vascular risk factors					
Smoker	149 (42.6)	117 (45.9)	32 (33.7)	4.212	0.040
Alcohol drinker	122 (34.9)	99 (38.8)	23 (24.2)	6.509	0.011
HT	255 (72.9)	191 (74.9)	64 (67.4)	1.986	0.159
DM	136 (38.9)	113 (44.3)	23 (24.2)	11.774	0.001
CHD	56 (16.0)	38 (14.9)	18 (18.9)	0.843	0.359
AF	36 (10.3)	25 (9.8)	11 (11.6)	0.236	0.627
DL	196 (56.0)	158 (62.0)	38 (40.0)	13.547	<0.001
Blood pressure					
SBP (mmHg)	145 (135-160)	150 (135-160)	140 (131-160)	-1.566	0.117
DBP (mmHg)	90 (80-100)	90 (80-100)	85 (80-100)	-1.592	0.111
Laboratory examination					
FBG (mmol/L)	5.99 (5.15-8.39)	6.21 (5.24-8.87)	5.58 (4.96-6.77)	-3.498	<0.001
TG (mmol/L)	1.57 (1.12-2.19)	1.72 (1.22-2.44)	1.19 (0.91-1.66)	-5.734	<0.001
TC (mmol/L)	4.77 (4.07-5.53)	4.84 (4.13-5.59)	4.61 (3.99-5.31)	-1.733	0.083
HDL-C (mmol/L)	1.14 (0.97-1.30)	1.11 (0.93-1.27)	1.16 (1.01-1.42)	-2.596	0.009
LDL-C (mmol/L)	2.72 (2.22-3.40)	2.84 (2.25-3.40)	2.62 (2.17-3.36)	-1.174	0.240
Post-admission treatment					
Antihypertensive	107 (30.6)	80 (31.4)	27 (28.4)	0.284	0.594
Anticoagulant	7 (2.0)	3 (1.2)	4 (4.2)	3.251	0.071
Antiplatelet agent	343 (98.0)	250 (98.0)	93 (97.9)	0.007	0.932
Statins	284 (81.1)	202 (79.2)	82 (86.3)	2.280	0.131
Involvement index					
TyG-BMI	231.22 (204.88-255.55)	240.02 (209.87-261.25)	208.53 (181.69-237.75)	-6.454	<0.001
TyG-WC	839.34 (765.69-913.91)	873.60 (800.71-931.87)	767.88 (677.79-837.35)	-7.596	<0.001

Values are expressed as median (Q1–Q3) or n (%).

### 3.3 Participants’ characteristics grouped by mRS score in the moderate-severe stroke group

As seen in **Table 3**, among 106 patients with moderate-to-severe stroke, 42 (39.6%) had good outcomes and 64 (60.4%) had poor outcomes. When comparing the two groups, there were statistically significant differences in gender, alcohol drinker, TG, HDL-C, TyG-BMI as well as TyG-WC (P <0.05). Compared with the good outcomes group, patients with poor outcomes had a more proportion of males and higher HDL-C, and a less proportion of alcohol drinkers, and lower TG, TyG-BMI, and TyG-WC (P <0.05).

**Table 3:** Participants’ characteristics grouped by mRS score in the moderate-severe stroke group.

Variables	Total (N = 106)	Good outcome group (N = 42)	Poor outcome group (N = 64)	Z/χ2	p
Demography					
Age (years)	67 (59-73)	68 (59-71)	67 (59-74)	-0.549	0.583
Gender (male)	66 (62.3)	32 (76.2)	34 (53.1)	5.742	0.017
Vascular risk factors					
Smoker	58 (54.7)	24 (57.1)	34 (53.1)	0.165	0.684
Alcohol drinker	39 (36.8)	21 (50.0)	18 (28.1)	5.218	0.022
HT	85 (80.2)	37 (88.1)	48 (75.0)	2.737	0.098
DM	36 (34.0)	15 (35.7)	21 (32.8)	0.095	0.758
CHD	21 (19.8)	9 (21.4)	12 (18.8)	0.115	0.735
AF	42 (39.6)	18 (42.9)	24 (37.5)	0.304	0.581
DL	40 (37.7)	15 (35.7)	25 (39.1)	0.121	0.728
Blood pressure					
SBP (mmHg)	160 (140-175)	150 (140-175)	160 (140-179)	-0.632	0.527
DBP (mmHg)	93 (80-105)	95 (84-106)	90 (80-104)	-0.770	0.442
Laboratory examination					
FBG (mmol/L)	5.90 (5.20-8.67)	6.54 (5.30-8.75)	5.70 (5.05-8.79)	-1.386	0.166
TG (mmol/L)	1.34 (0.95-1.73)	1.41 (1.16-1.99)	1.23 (0.78-1.70)	-2.154	0.031
TC (mmol/L)	4.63 (4.09-5.18)	4.64 (4.14-5.10)	4.55 (4.03-5.31)	-0.484	0.628
HDL-C (mmol/L)	1.16 (0.99-1.34)	1.08 (0.95-1.27)	1.19 (1.03-1.42)	-2.268	0.023
LDL-C (mmol/L)	2.63 (2.21-3.11)	2.65 (2.36-3.18)	2.60 (2.09-2.99)	-0.759	0.448
Post-admission treatment					
Antihypertensive	43 (40.6)	16 (38.1)	27 (42.2)	0.176	0.675
Anticoagulant	2 (1.9)	0 (0.0)	2 (3.1)	1.338	0.247
Antiplatelet agent	104 (98.1)	41 (97.6)	63 (98.4)	0.092	0.762
Statins	82 (77.4)	30 (71.4)	52 (81.3)	1.397	0.237
Involvement index					
TyG-BMI	211.55 (191.05-243.28)	225.53 (208.37-260.35)	205.76 (174.51-230.41)	-3.740	<0.001
TyG-WC	761.72 (689.83-837.48)	811.81 (740.44-879.33)	735.80 (658.13-805.24)	-3.520	<0.001

Values are expressed as median (Q1–Q3) or n (%).

### 3.4 Participants’ characteristics divided by TyG-BMI quartile

As seen in **Table 4**, based on TyG-BMI quartiles, patients were grouped into four groups (Q1, Q2, Q3, Q4), each comprising 114 patients. When comparing the four groups, there were statistically significant differences in age, HT, DM, DL, FBG, TG, TC, HDL-C, and LDL-C (P <0.05). Compared with the lower quartile groups, the higher quartile groups had a more proportion of HT, DM, DL, and higher FBG, TG, TC, and LDL-C, and lower age and HDL-C (P <0.05).

**Table 4:** Characteristics of participants stratified by TyG-BMI quartile.

Variables	Q1	Q2	Q3	Q4	Z/χ2	p
	110.65 - 200.81	201.19 - 225.14	225.23 - 254.15	254.16 - 370.00		
Demography						
Age (years)	67 (60-75)	65 (58-72)	65 (56-68)	62 (56-69)	18.027	<0.001
Gender (male)	70 (61.4)	78 (68.4)	75 (65.8)	72 (63.2)	1.411	0.703
Vascular risk factors						
Smoker	58 (50.9)	57 (50.0)	41 (36.0)	51 (44.7)	6.467	0.091
Alcohol drinker	37 (32.5)	48 (42.1)	35 (30.7)	41 (36.0)	3.792	0.285
HT	77 (67.5)	79 (69.3)	97 (85.1)	87 (76.3)	11.469	0.009
DM	16 (14.0)	38 (33.3)	51 (44.7)	67 (58.8)	52.052	<0.001
CHD	23 (20.2)	19 (16.7)	16 (14.0)	19 (16.7)	1.547	0.671
AF	19 (16.7)	24 (21.1)	19 (16.7)	16 (14.0)	2.042	0.564
DL	36 (31.6)	52 (45.6)	69 (60.5)	79 (69.3)	37.871	<0.001
Blood pressure						
SBP (mmHg)	142 (130-160)	150 (135-164)	150 (140-165)	150 (135-170)	5.650	0.130
DBP (mmHg)	85 (80-100)	90 (80-100)	90 (80-100)	90 (80-100)	5.883	0.117
Laboratory examination						
FBG (mmol/L)	5.18 (4.87-5.80)	5.68 (5.15-7.44)	6.46 (5.36-8.90)	7.86 (5.89-10.89)	95.128	<0.001
TG (mmol/L)	1.02 (0.75-1.42)	1.35 (1.08-1.74)	1.76 (1.41-2.35)	2.01 (1.49-3.19)	132.224	<0.001
TC (mmol/L)	4.43 (3.80-5.01)	4.71 (4.07-5.47)	4.76 (4.15-5.51)	5.01 (4.35-5.72)	18.601	<0.001
HDL-C (mmol/L)	1.22 (1.03-1.45)	1.15 (0.98-1.31)	1.10 (0.91-1.24)	1.10 (0.96-1.26)	20.026	<0.001
LDL-C (mmol/L)	2.43 (2.01-2.99)	2.74 (2.31-3.20)	2.78 (2.27-3.28)	3.01 (2.39-3.61)	19.362	<0.001

Values are expressed as median (Q1–Q3) or n (%).

### 3.5 Participants’ characteristics stratified by TyG-WC quartile

As seen in **Table 5**, based on TyG-WC quartiles, patients were separated into four groups (Q1, Q2, Q3, Q4), each comprising 114 patients. When comparing the four groups, there were statistically significant differences in age, gender, smoker, alcohol drinker, HT, DM, DL, FBG, TG, TC, HDL-C, and LDL-C (P <0.05). Compared with the lower quartile groups, the higher quartile groups had a more proportion of males, smokers, alcohol drinkers, HT, DM, DL, and higher FBG, TG, TC, and LDL-C, and lower age and HDL-C (P <0.05).

**Table 5:** Characteristics of participants stratified by TyG-WC quartile.

Variables	Q1	Q2	Q3	Q4	Z/χ2	P
	491.26 - 742.82	742.83 - 828.47	829.53 - 900.56	901.79 - 1277.63		
Demography						
Age (years)	66 (60-74)	65 (56-72)	65 (58-72)	62 (54-68)	18.149	<0.001
Gender (male)	60 (52.6)	76 (66.7)	73 (64.0)	86 (75.4)	13.240	0.004
Vascular risk factors						
Smoker	56 (49.1)	54 (47.4)	38 (33.3)	59 (51.8)	9.369	0.025
Alcohol drinker	33 (28.9)	46 (40.4)	33 (28.9)	49 (43.0)	8.247	0.041
HT	79 (69.3)	78 (68.4)	86 (75.4)	97 (85.1)	10.637	0.014
DM	14 (12.3)	27 (23.7)	53 (46.5)	78 (68.4)	90.438	<0.001
CHD	24 (21.1)	15 (13.2)	18 (15.8)	20 (17.5)	2.672	0.445
AF	25 (21.9)	16 (14.0)	22 (19.3)	15 (13.2)	4.269	0.234
DL	37 (32.5)	45 (39.5)	69 (60.5)	85 (74.6)	51.151	<0.001
Blood pressure						
SBP (mmHg)	150 (135-175)	140 (135-160)	150 (135-160)	150 (140-170)	7.287	0.063
DBP (mmHg)	90 (80-100)	85 (80-95)	90 (80-95)	90 (80-100)	7.110	0.068
Laboratory examination						
FBG (mmol/L)	5.26 (4.87-5.66)	5.59 (5.01-6.28)	6.55 (5.53-8.61)	8.98 (6.57-12.00)	129.940	<0.001
TG (mmol/L)	0.96 (0.74-1.30)	1.43 (1.12-1.77)	1.67 (1.37-2.18)	2.20 (1.61-3.39)	170.437	<0.001
TC (mmol/L)	4.43 (3.89-4.98)	4.74 (4.02-5.36)	4.83 (4.22-5.60)	5.06 (4.27-5.65)	18.285	<0.001
HDL-C (mmol/L)	1.24 (1.06-1.48)	1.16 (0.98-1.28)	1.10 (0.94-1.27)	1.07 (0.91-1.23)	28.522	<0.001
LDL-C (mmol/L)	2.43 (2.03-3.05)	2.74 (2.22-3.28)	2.86 (2.40-3.39)	2.86 (2.27-3.46)	15.992	0.001

Values are expressed as median (Q1–Q3) or n (%).

### 3.6 Correlation of TyG-BMI and TyG-WC with severity of acute ischemic stroke

As seen in **Table 6**, the severity of new-onset acute ischemic stroke was used as the dependent variable (moderate-severe = 1, mild = 0), and the independent variable was TyG-BMI (the quartile 1 group served as the reference group). Three models were employed in a multivariate logistic regression analysis of the data. Without adjusting for any variables (Model 1), quartiles 3 and 4 of TyG-BMI had sequentially lower risk of moderate-severe stroke (P <0.05); after adjusting for age (Model 2), the above patterns remained relevant (P <0.05); After adjusting for age, smoker, AF, DL, SBP, DBP, and TG (Model 3), quartile 4 of TyG-BMI had a reduced risk of moderate-severe stroke (P <0.05).

**Table 6:** Logistic regression analysis of TyG-BMI and stroke severity.

Variables	Model 1			Model 2			Model 3		
	OR	95%CI	P	OR	95%CI	P	OR	95%CI	P
Q1	1.000 (Ref)			1.000 (Ref)			1.000 (Ref)		
Q2	0.846	(0.479-1.492)	0.563	0.881	(0.497-1.562)	0.665	0.779	(0.404-1.499)	0.454
Q3	0.461	(0.247-0.860)	0.015	0.496	(0.264-0.934)	0.030	0.489	(0.234-1.021)	0.057
Q4	0.406	(0.214-0.770)	0.006	0.443	(0.231-0.848)	0.014	0.407	(0.185-0.894)	0.025

Model 1, unadjusted for confounders. Model 2, adjusted age; Model 3, adjusted age, smoker, AF, DL, SBP, DBP, and TG. OR, odds ratio; CI, confidence interval; Ref, Reference.

As seen in **Table 7**, the severity of new-onset acute ischemic stroke was used as the dependent variable (all assignments and reference Settings were the same as above), and the independent variable was TyG-WC. Three models were employed in a multivariate logistic regression analysis of the data. Without adjusting for any variables (Model 1), quartiles 2, 3, and 4 of TyG-WC had sequentially lower risk of moderate-severe stroke (P <0.05); after adjusting for age (Model 2), the above patterns remained relevant (P <0.05); After adjusting for age, smoker, AF, DL, SBP, DBP, and TG (Model 3), quartiles 3 and 4 of TyG-WC had a reduced risk of moderate-severe stroke (P <0.05).

**Table 7:** Logistic regression analysis of TyG-WC and stroke severity.

Variables	Model 1			Model 2			Model 3		
	OR	95%CI	P	OR	95%CI	P	OR	95%CI	P
Q1	1.000 (Ref)			1.000 (Ref)			1.000 (Ref)		
Q2	0.523	(0.297-0.920)	0.024	0.546	(0.309-0.964)	0.037	0.641	(0.334-1.232)	0.182
Q3	0.326	(0.177-0.601)	<0.001	0.333	(0.180-0.615)	<0.001	0.355	(0.173-0.728)	0.005
Q4	0.180	(0.089-0.366)	<0.001	0.195	(0.095-0.399)	<0.001	0.140	(0.056-0.351)	<0.001

Model 1, unadjusted for confounders. Model 2, adjusted age; Model 3, adjusted age, smoker, AF, DL, SBP, DBP, and TG. OR, odds ratio; CI, confidence interval; Ref, Reference.

### 3.7 Correlation of TyG-BMI and TyG-WC with short-term outcome of acute ischemic stroke

As seen in **Table 8**, short-term outcomes of new-onset acute ischemic stroke were used as the dependent variable (poor outcome = 1, good outcome = 0), and the independent variable was TyG-BMI (with the quartile 1 group as the reference). Three models were employed in a multivariate logistic regression analysis of the data. Without adjusting for any variables (Model 1), quartiles 2, 3, and 4 of TyG-BMI had a reduced risk of adverse outcomes (P <0.05); after adjusting for gender and age (Model 2), the results did not change significantly (P <0.05); These patterns remained relevant after adjusting for confounding variables like gender, age, smoker, alcohol drinker, DM, DL, FBG, TG, HDL-C, and stroke severity (Model 3) (P <0.05).

**Table 8:** Logistic regression analysis of TyG-BMI and short-term outcome of stroke.

Variables	Model 1			Model 2			Model 3		
	OR	95%CI	P	OR	95%CI	P	OR	95%CI	P
Q1	1.000 (Ref)			1.000 (Ref)			1.000 (Ref)		
Q2	0.328	(0.191-0.562)	<0.001	0.337	(0.193-0.587)	<0.001	0.394	(0.215-0.722)	0.003
Q3	0.208	(0.118-0.366)	<0.001	0.206	(0.114-0.371)	<0.001	0.324	(0.163-0.642)	0.001
Q4	0.095	(0.050-0.183)	<0.001	0.089	(0.045-0.175)	<0.001	0.158	(0.027-0.349)	<0.001

Model 1, unadjusted for confounders. Model 2, adjusted gender and age; Model 3, adjusted gender, age, smoker, alcohol drinker, DM, DL, FBG, TG, HDL-C, and Stroke severity. OR, odds ratio; CI, confidence interval; Ref, Reference.

As seen in **Table 9**, short-term outcomes of new-onset acute ischemic stroke were the dependent variable and TyG-WC was the independent variable (all assignments and reference settings are the same as above). Three models were employed in a multivariate logistic regression analysis of the data. Without adjusting for any variables (Model 1), quartiles 2, 3, and 4 of TyG-WC had sequentially lower risk of short-term adverse outcomes (P <0.05); after adjusting for gender and age (Model 2), the above patterns remained relevant (P <0.05); after adjusting for gender, age, smoker, alcohol drinker, DM, DL, FBG, TG, HDL-C, and stroke severity (Model 3), quartiles 3 and 4 of TyG-WC had a reduced risk of short-term adverse outcomes (P <0.05).

**Table 9:** Logistic regression analysis of TyG-WC and short-term outcome of stroke.

Variables	Model 1			Model 2			Model 3		
	OR	95%CI	P	OR	95%CI	P	OR	95%CI	P
Q1	1.000 (Ref)			1.000 (Ref)			1.000 (Ref)		
Q2	0.395	(0.231-0.673)	0.001	0.441	(0.256-0.762)	0.003	0.632	(0.347-1.151)	0.134
Q3	0.199	(0.113-0.351)	<0.001	0.208	(0.117-0.371)	<0.001	0.350	(0.175-0.700)	0.003
Q4	0.069	(0.034-0.139)	<0.001	0.081	(0.039-0.167)	<0.001	0.178	(0.071-0.451)	<0.001

Model 1, unadjusted for confounders. Model 2, adjusted gender and age; Model 3, adjusted gender, age, smoker, alcohol drinker, DM, DL, FBG, TG, HDL-C, and Stroke severity. OR, odds ratio; CI, confidence interval; Ref, Reference.

## 4 Discussion

The findings of this cross-sectional investigation revealed that TyG-BMI as well as TyG-WC were correlated with initial neurological impairment and short-term outcomes with new-onset ischemic stroke to varying degrees.

In recent years, there has been growing evidence that insulin resistance (IR) plays a significant role in obesity, ischemic stroke, and abnormalities of glucose and lipid metabolism, and is a common pathological feature of the above diseases (28). IR is associated with atherosclerosis through metabolic abnormalities. As a simple replacement for IR, TyG is closely linked to homeostatic model assessment (HOMA-IR) as well as insulin-stimulated glucose uptake (29). A nationwide Chinese study discovered that the index for TyG possessed a significant degree of sensitivity and specificity in identifying people with vascular disease and metabolic disorders (30). In addition, BMI and WC combined with TyG had better efficacy in predicting the occurrence of ischemic stroke. A cohort investigation of 9,406 participants showed that TyG-BMI and TyG-WC were more predictive of ischemic stroke than TyG (18).

There are no studies examining the effects of TyG-WC and TyG-BMI on initial neurologic deficits and short-term prognosis in ischemic stroke. However, it has been demonstrated that higher TG and greater WC are linked to milder stroke outcomes (7, 8, 31). A study with 12,964 individuals found that overweight and obese stroke patients had considerably reduced mortality than patients with normal BMI (32). Kang et al. discovered that increased waist circumference at admission was linked with less severe stroke (8). Consistent with the above results, the present research found that higher TyG-WC and TyG-BMI were followed by lower severity and better short-term outcomes in stroke. This contrasts with the general perception that obesity increases the risk of disease. Many reports have demonstrated that obesity has a favorable effect on the prognosis of individuals with stroke, an occurrence known as the stroke-obesity paradox (33).

Research suggests that one explanation in terms of physiology is that the obesity paradox may be the result of a physiological process in which two characteristics associated with obesity are interrelated. Endogenous glucose production (EGP) is one example. Obesity is connected with increased EGP capacity and thus increased glucose availability (34). Previous experiments have shown that glucose prioritizes the immune system’s needs, sustains macrophage viability, and slows disease development (35). The other factor is insulin resistance, which limits the quantity of glucose that non-obligate glucose consumers can metabolize while increasing the production of glycerol and fatty acids through the fat pool as an alternate fuel source for tissues and cells (36). Although it is not clear whether IR is involved in the stroke-obesity paradox, IR theoretically contributes to this phenomenon. Body mass index and *in vivo* homeostasis model assessment-insulin resistance have been proven to have a substantial relationship on the probability of death and poor functional prognosis (12).

At present, the obesity paradox has been proven to exist in a variety of diseases, including acute myocardial infarction, stroke, coronary heart disease (37), and so on. But still, some scholars are skeptical about it. Some studies have suggested that the obesity paradox in stroke is not true and may be due to selection bias (38). Recent researches have shown that the obesity paradox is not universal and that a variety of variables such as gender, uric acid levels, and insulin sensitivity influence its existence (39, 40). One methodological interpretation of the obesity paradox is that it is partly due to a selective bias, which is called “collider stratification bias” (41). When exposure variables and disease are considered in the same analysis, selection bias arises. Stated differently, it is the result of the action of conditions that are jointly affected by exposure factors and disease outcomes (38). Many scholars have demonstrated that by selecting populations based on collider variables, spurious associations between exposures and outcomes may be created, and even the positive and negative direction of the correlation can be reversed, making exposures that are detrimental to outcomes appear to have a beneficial effect (38, 42). Collider variables are variables for which the variable risk and the associated outcome have at least two common causes, and such interfering variables include lifestyle and unmeasured genetic factors such as genes (43, 44). For example, obese patients coexist with a family history of hypercholesterolemia, and individuals at greatest genetic risk gain greater clinical benefit from statin therapy (45, 46), so this obesity paradox of interference by genetic factors is not true.

In this study, higher TyG-WC and TyG-BMI patients were found to be more likely to use lipid-lowering, antihypertensive, and antiplatelet medications in the usual way in the data statistics. Therefore, this study excluded patients who used medications before admission and adjusted for patient characteristics including age, gender, smoker, alcohol drinker, DM, AF, DL, SBP, DBP, FBG, TG, and HDL-C.

Despite adjusting for these potential confounders, the obesity paradox persisted. We speculate that part of the reason for the outcome of this study may be related to the socioeconomic status of the patients. Higher income families are more likely to have overweight or obese members, and at the same time, they can receive timely and effective treatment after the diagnosis of stroke. Another reason may be that obese and overweight patients can contribute to vascular arteriosclerosis, with some patients progressively developing stenosis or obstruction of blood vessels (47). To adapt to this change, collateral circulation will be established compensatively. In patients with good collateral circulation, once blood vessels are occluded and thrombosis is formed, collateral vessels will provide part of the blood supply, leading to slow brain tissue ischemia, less necrosis of brain cells, and relatively mild neurological function defect. In addition, studies have shown that a family history of stroke is an important risk factor for obesity and hyperlipidemia (48, 49), and these patients may also have cardiovascular and metabolic diseases. They will have active health improvement behaviors. In the event of stroke, they also know how to receive effective treatment within the time window, reducing delays in pre-hospital and in-hospital conditions, thereby reducing the amount of necrotic brain cells and thus the degree of neurological impairment (50).

This study has several drawbacks as well. First, because this was a retrospective investigation, a causal association between the TyG combined with obesity index and initial neurological severity and short-term prognosis of acute ischemic stroke could not be inferred. Second, this study did not provide lengthy follow-up of stroke patients, therefore the association between the indices involved and the long-term prognosis of individuals with acute ischemic stroke is unknown. In addition, the data of this study were limited in that only the Chinese population was analyzed, and the results may not be applicable to other ethnic groups.

## 5 Conclusions

In summary, this study demonstrated that TyG-WC as well as TyG-BMI were correlated with the severity and short-term outcome of new-onset ischemic stroke to varying degrees. As their quartile grouping increased, the degree of initial neurological deficits in stroke decreased and the short-term prognosis was better.

## Data availability statement

The raw data supporting the conclusions of this article will be made available by the authors, without undue reservation.

## Ethics statement

The studies involving humans were approved by The Affiliated Hospital of Beihua University’s Medical Ethics Committee. The studies were conducted in accordance with the local legislation and institutional requirements. The participants provided their written informed consent to participate in this study.

## Author contributions

X-RY: Conceptualization, Data curation, Investigation, Methodology, Writing – original draft, Writing – review & editing. J-LD: Data curation, Writing – review & editing. MJ: Data curation, Writing – review & editing. YR: Data curation, Writing – review & editing. F-LZ: Writing – review & editing. F-LK: Funding acquisition, Writing – review & editing. F-EL: Conceptualization, Funding acquisition, Methodology, Supervision, Writing – review & editing.
